# Speech serial control in healthy speakers and speakers with hypokinetic or ataxic dysarthria: effects of sequence length and practice

**DOI:** 10.3389/fnhum.2013.00665

**Published:** 2013-10-16

**Authors:** Kevin J. Reilly, Kristie A. Spencer

**Affiliations:** ^1^Department of Speech-Language Pathology and Audiology, Northeastern UniversityBoston, MA, USA; ^2^Department of Speech and Hearing Sciences, University of WashingtonSeattle, WA, USA

**Keywords:** speech production, sequence length, sequence learning, Parkinson’s disease, hypokinetic dysarthria, ataxic dysarthria, basal ganglia, cerebellum

## Abstract

The current study investigated the processes responsible for selection of sounds and syllables during production of speech sequences in 10 adults with hypokinetic dysarthria from Parkinson’s disease, five adults with ataxic dysarthria, and 14 healthy control speakers. Speech production data from a choice reaction time task were analyzed to evaluate the effects of sequence length and practice on speech sound sequencing. Speakers produced sequences that were between one and five syllables in length over five experimental runs of 60 trials each. In contrast to the healthy speakers, speakers with hypokinetic dysarthria demonstrated exaggerated sequence length effects for both inter-syllable intervals (ISIs) and speech error rates. Conversely, speakers with ataxic dysarthria failed to demonstrate a sequence length effect on ISIs and were also the only group that did not exhibit practice-related changes in ISIs and speech error rates over the five experimental runs. The exaggerated sequence length effects in the hypokinetic speakers with Parkinson’s disease are consistent with an impairment of action selection during speech sequence production. The absent length effects observed in the speakers with ataxic dysarthria is consistent with previous findings that indicate a limited capacity to buffer speech sequences in advance of their execution. In addition, the lack of practice effects in these speakers suggests that learning-related improvements in the production rate and accuracy of speech sequences involves processing by structures of the cerebellum. Together, the current findings inform models of serial control for speech in healthy speakers and support the notion that sequencing deficits contribute to speech symptoms in speakers with hypokinetic or ataxic dysarthria. In addition, these findings indicate that speech sequencing is differentially impaired in hypokinetic and ataxic dysarthria.

## INTRODUCTION

Accurate sequencing of phonetic and phonologic content is a critical aspect of fluent speech production (Lashley, 1951). Analyses of speech errors and chronometric variations have provided compelling evidence that the representations of most or all of the items in a to-be-produced speech sequence are activated, or available for production, prior to the initiation of that sequence (Lashley, 1951; MacKay, 1970; Sternberg et al., 1978, 1980; Shattuck-Hufnagel, 1979; Gordon and Meyer, 1987; Klapp, 2003). As a result, models of speech sound sequencing typically include a working memory or response buffer into which retrieved speech items are loaded, and a selection process that chooses, from among the items, the one that is to be produced next (Stemberger, 1985; Dell, 1986, 1988; Hartley and Houghton, 1996; Bohland et al., 2010). To date, chronometric investigations have focused largely on speech reaction times, which reflect processes related to buffer loading and selection of a sequence-initial item. In contrast, there are few studies of within-sequence chronometric measures that reflect the processes by which the selection of successive speech sounds and syllables unfolds during sequence production. The lack of corresponding within-sequence chronometric datasets ignores a potentially rich and diagnostically useful aspect of sequence performance. The current study evaluated speech errors and within-sequence chronometric measures to investigate the processes that support the selection of speech sounds and syllables during sequence production in healthy speakers and speakers with dysarthria related to either cerebellar damage (i.e., ataxic dysarthria) or Parkinson’s disease (i.e., hypokinetic dysarthria). Results of these analyses were used to determine whether serial control deficits contribute to the speech symptomatology observed in these clinical populations.

### THE EFFECTS OF SEQUENCE LENGTH AND PRACTICE ON SPEECH SEQUENCING

A benchmark assessment of the integrity of speech sequencing ability is the demonstration of “sequence length effects.” These effects describe the systematic and predictable changes in reaction time and production rate that occur with changes to the length of the forthcoming movement sequence. For example, speech reaction time studies have robustly demonstrated that increases in the length of a to-be-produced sequence increase the reaction time to initiate a sequence (Klapp, 1974; Sternberg et al., 1978, 1980; Peters et al., 1989; Jared and Seidenberg, 1990; Deger and Ziegler, 2002; Roelofs, 2002; Santiago et al., 2002; Spencer and Rogers, 2005) and also increase the time interval between successive sequence items (i.e., the inter-response interval; Sternberg et al., 1978; Klapp, 2003). Increases in sequence length have also been associated with increases in speech error rates (Sternberg et al., 1980; Jared and Seidenberg, 1990). The effects of sequence length on error and chronometric measures have also been reported during production of non-speech sequences (Sternberg et al., 1978; Rosenbaum et al., 1984b; Rafal et al., 1987; Canic and Franks, 1989; Verwey, 1994; Verwey and Eikelboom, 2003), which suggests that length effects reflect aspects of serial control that are common across movement modalities. Reaction time effects have been attributed to the increased time required to buffer additional items in a sequence (Sternberg et al., 1978; Rosenbaum et al., 1984a) and to select the initial sequence item from among a greater number of competing items (Boardman and Bullock, 1991; Bullock and Rhodes, 2003; Rhodes et al., 2004). In contrast, sequence length effects on inter-response intervals have been associated with processes underlying ordered selection of sequence items within the response buffer (Boardman and Bullock, 1991; Klapp, 1995; Hartley and Houghton, 1996; Page and Norris, 1998; Bullock and Rhodes, 2003; Rhodes et al., 2004). Specifically, increasing the number of sequence items increases the time to select the next item in the sequence and reduces the likelihood of selecting the correct item. In the current study, sequence length effects on inter-syllable intervals (ISIs) and speech error rates were evaluated to delineate the dynamics of the selection process during speech sequencing in healthy speakers and speakers with dysarthria secondary to Parkinson’s disease or cerebellar damage.

A second aspect of the current study addressed the effects of practice on speech sequencing performance in healthy and neurologic populations. Reaction time investigations have revealed that practicing a novel sequence or set of sequences increases performance accuracy, decreases reaction times, and decreases inter-response intervals. These effects have been observed during production of both speech (MacKay, 1982; Klapp, 2003; Smits-Bandstra et al., 2006) and non-speech (MacKay, 1982; Klapp, 2003; Smits-Bandstra et al., 2006) movement sequences. One aspect of sequence learning involves a compressive recoding of multiple sequence items into a single item, or chunk (Brown and Carr, 1989; van Mier et al., 1993; Klapp, 1995, 2003; Verwey, 1996; Sakai et al., 2003), that thereby increases the efficiency of serial processing. Chunking of sequences items allows for fast parallel loading of sequence items into a response buffer and can account for the reduced reaction times associated with practiced sequences. Given the over-learned nature of speech production (Netsell, 1982), chunk formation probably accounts for the fluent production of many sound and syllable combinations during speech production. A second mechanism involves contextual learning of item-by-item transitions within a sequence such that the selection of a sequence item immediately facilitates the selection of the following item in a sequence. This next-item facilitation accelerates the transitions between items in a sequence and has been linked to the decreased inter-response intervals observed during production of practice sequences (Rhodes and Bullock, 2002a, b; Rhodes et al., 2004). The current investigation extends the findings of previous speech studies to evaluate practice effects over multiple presentations of a larger number of sequences varying in length between one and five syllables.

### SERIAL CONTROL IN SPEAKERS WITH HYPOKINETIC DYSARTHRIA FROM PARKINSON’S DISEASE

Neuroimaging investigations indicate that serial control for speech is distributed across a network of cortical and subcortical structures including the basal ganglia and cerebellum (Riecker et al., 2005, 2006, 2008; Bohland and Guenther, 2006; Klein et al., 2006; Soros et al., 2006; Peeva et al., 2010). In addition, impairments in the production of fluent speech sequences have been reported in speakers with damage to the cerebellum (Netsell and Kent, 1976; Kent et al., 1997; Riva, 1998; Ackermann et al., 2004) and basal ganglia (Fabbro et al., 1996; Pickett et al., 1998; Kreisler et al., 2000; Goberman et al., 2010). The current study investigated speech selection processes during sequence production in speakers with dysarthria related to either Parkinson’s disease (i.e., hypokinetic dysarthria) or cerebellar damage (i.e., ataxic dysarthria).

Parkinson’s disease is a neurodegenerative disorder resulting from depletion of dopamine levels in the basal ganglia. In addition to the classical motor symptoms, nearly 90% of individuals with Parkinson’s disease will develop speech and swallowing disorders during the course of their illness (Logemann et al., 1978; Hartelius and Svensson, 1994; Ho et al., 1998). The speech impairment of Parkinson’s disease, or hypokinetic dysarthria, is thought by some to emerge from a combination of motor execution deficiencies (e.g., the effects of rigidity) as well as motor programing deficits (Van der Merwe, 1997; Spencer and Rogers, 2005; Skodda et al., 2010). The speech symptoms that align with a disruption to the motor programing of sequences include speech rate difficulties (Van der Merwe, 1997), stopping an ongoing response, marked hesitations between syllables, abnormally placed pauses, and difficulty with progression through an utterance (Spencer and Rogers, 2005; Spencer, 2007).

Contemporary accounts of basal ganglia function highlight the importance of these nuclei in facilitating the selection of a desired action plan from a number of alternative competing plans (Mink, 1996; Kropotov and Etlinger, 1999; Redgrave et al., 1999; Brown et al., 2004). In these accounts, the striatum acts as a “competitive arena” (Brown et al., 2004) wherein cortical plan representations excite striatal spiny projection neurons corresponding to a particular action plan and inhibit spiny projection neurons of competing plans via GABA-ergic striatal interneurons (Brown et al., 2004; Bullock and Tan, 2007). Striatal competition among the action plans ultimately leads to disinhibition of a “winning” plan (i.e., the most “salient” or urgent action plan), which is then made available for production (Mink, 1996; Brown et al., 2004; Wiecki and Frank, 2010; Frank, 2011). The timing and accuracy of this selection process depend largely on the degree of contrast between the activation level of the salient or higher priority action plan and the activation levels of background, competing plans. Specifically, greater contrast reduces the time of the competition to select a “winner” and also reduces the likelihood of a selection error. Of relevance to the current study is the finding that the total activity across sequence items is conserved (Averbeck et al., 2002; Cisek and Kalaska, 2002) and, as a result, increases in the number of items has the effect of reducing the contrast between those items. The reduced contrast, in turn, increases the time to select each “winning” item in a sequence. In addition, reduced contrast increases the likelihood of a selection error as the outcome of competition becomes increasingly sensitive to the inherent noise levels within the network. In this way, advances in basal ganglia physiology have led to the development of neural models of sequencing that are capable of accounting for the effects of sequence length on both speech error rates and inter-response intervals (Rhodes et al., 2004). In addition, these findings highlight the sensitivity of sequence length effects to the contrast in activation levels between competing items in a sequence.

Considerable evidence indicates that one aspect of dopamine function in the basal ganglia involves the facilitation of action selection by enhancing the contrast between the activation level of the salient or higher priority action plan and the activation levels of the background, competing plans (Foote and Morrison, 1987; Cohen and Servan-Schreiber, 1992; Kiyatkin and Rebec, 1996; Hernandez-Lopez et al., 1997; Rebec, 1997; Boraud et al., 2000; Cohen et al., 2002; Leblois et al., 2006). Dopamine depletion, in turn, reduces the contrast between plan activations in the striatum and, as a result, prolongs the time to select each “winning” item and increases the likelihood of a selection error (Brown and Robbins, 1989; Robbins et al., 1990; McGuire et al., 1996; Jurgens, 2002; Featherstone and McDonald, 2005; Fukabori et al., 2012; Nishizawa et al., 2012). Support for the effects of dopaminergic function on activation contrasts derives from the numerous findings of action-selection deficits in patients with Parkinson’s disease (Bloxham et al., 1987; Sheridan et al., 1987; Robertson and Flowers, 1990; Agostino et al., 1992; Jahanshahi et al., 1992, 1993; Brown et al., 1993; Gueye et al., 1998; Praamstra and Plat, 2001; Pollux, 2004; Wylie et al., 2005) and, in particular, on action selection during movement sequences (Benecke et al., 1987; Agostino et al., 1992; Georgiou et al., 1994). Of particular relevance is the finding that inter-response intervals become disproportionately longer, and error rates disproportionately higher, toward the ends of longer sequences in patients with Parkinson’s disease (Benecke et al., 1987; Agostino et al., 1992; Georgiou et al., 1994). These findings indicate that patients with Parkinson’s disease are more sensitive to the effects of sequence length on inter-response intervals and error rates. The possibility of exaggerated length effects in individuals with Parkinson’s disease is consistent with findings regarding the effects of dopamine depletion on the contrasts in activation levels between competing items in the striatum (Foote and Morrison, 1987; Cohen and Servan-Schreiber, 1992; Kiyatkin and Rebec, 1996; Rebec, 1997; Boraud et al., 2000; Cohen et al., 2002; Leblois et al., 2006). This reduction in contrast would augment the already reduced contrast across items in longer sequences and, as a result, increases the effects of sequence length on inter-response intervals and error rates. The possibility of exaggerated or larger-than-normal sequence length effects was evaluated in the current investigation, which compared sequence length effects on ISIs and speech error rates in speakers with dysarthria from Parkinson’s disease to those of neurologically healthy speakers and speakers with ataxic dysarthria.

### SERIAL CONTROL IN SPEAKERS WITH ATAXIC DYSARTHRIA

Damage to the cerebellum or its input and output pathways can give rise to a set of speech deficits known as ataxic dysarthria. Lesions studies have shown that ataxic dysarthria tends to result from right-sided cerebellar lesions (Ackermann et al., 1992; Amarenco et al., 1993; Urban et al., 2001) and especially right superior cerebellar lesions (Ackermann et al., 1992; Urban et al., 2001). Although speech deficits in ataxic dysarthria are generally thought to reflect speech execution impairments, recent evidence suggests that many of the cerebellar regions implicated in ataxic dysarthria are, through connections with the cerebral cortex, part of a phonologic working memory or buffering system for pre-loading to-be-produced items in a sequences (Desmond et al., 1997; Ackermann et al., 2004; Chen and Desmond, 2005; Kirschen et al., 2005; Bohland and Guenther, 2006; Spencer and Slocomb, 2007; Strick et al., 2009; Durisko and Fiez, 2010; Marvel and Desmond, 2010b, 2010a, 2012). As a result, it has been suggested that a portion of the symptoms associated with ataxic dysarthria may reflect disruptions to the processing buffers responsible for speech motor programing of speech sequences (Van der Merwe, 1997; Spencer and Rogers, 2005; Spencer and Slocomb, 2007). In particular, deviant symptoms of ataxic dysarthria, such as the “scanning” speech quality, disrupted rate and rhythm (Kent et al., 2000), and timing irregularities (Kent et al., 1997) are all compatible with the premise that an impairment in the motor programing of speech sequences in ataxic dysarthria is disrupted in addition to speech execution. In particular, Kent et al. (1997) noted that difference in speech performance between healthy speakers and speakers with ataxic dysarthria more apparent during production of repeated syllables at fast versus slow rates.

Evidence that impairments to processing buffers in cerebellar ataxia can affect serial control was provided by Inhoff et al. (1989). In their investigation of patients with cerebellar lesions, patients with bilateral lesions and moderate, but not mild, symptoms of ataxia failed to exhibit the expected sequence length effects on reaction times and inter-response intervals during performance of a sequential keypress task. In addition, patients with unilateral cerebellar lesions and moderate ataxia exhibited sequence length effects on the unaffected side (i.e., contralateral to the lesion) but not on the affected side (i.e., ipsilateral to the lesion). The authors concluded that the absent length effects in the moderately ataxic patients indicated a limited capacity to pre-load sequence items. As a result, only one or more earlier-occurring items in a sequence were available for selection during sequence production and later-occurring items in the sequence were loaded during production of the earlier items. In a similar study involving speech production, Spencer and Rogers (2005) examined sequence length effects on speech reaction times during production of non-sense syllables that varied in length from one to five syllables. These investigators observed that speakers with ataxic dysarthria exhibited reduced sequence length effects on speech reaction times compared to the healthy control speakers. Together, the findings of Inhoff et al. (1989) and Spencer and Rogers (2005) suggest that the cerebellum is involved in “pre-loading” or buffering movement plans prior to production of a movement sequence. The current study extended the reaction time findings of the Spencer and Rogers (2005) study and evaluated the effects of sequence length on ISIs in the sequence productions of speakers in that study.

Investigations of sequence learning for non-speech sequences have found that patients with cerebellar disease fail to exhibit the expected decreases in error rate and inter-response intervals typically associated sequence practice (Pascual-Leone et al., 1993; Molinari et al., 1997; Gomez-Beldarrain et al., 1998). These findings are in agreement with numerous investigations that implicate cerebellar processing in both sequence learning (Jenkins et al., 1994; Toni et al., 1998; Steele and Penhune, 2010) and retrieval of learned sequences (Lu et al., 1998; Wu et al., 2008; Steele and Penhune, 2010). The current study evaluated whether the reduced effects of practice in cerebellar patients during production of non-speech sequences is also evident during production of speech sequences by speakers with ataxic dysarthria. This possibility was evaluated by comparing practice-related changes in ISIs and error rates produced by the healthy control and hypokinetic speaker groups to those produced by the ataxic speaker group.

## MATERIALS AND METHODS

### PARTICIPANTS

Data for the current study consisted of speech sequence productions from Experiment 2 of Spencer and Rogers (2005). The participants were 10 adults with hypokinetic dysarthria (mean age = 64.1, SD = 14.2), five adults with ataxic dysarthria (mean age = 30.0, SD = 6.2), and 15 healthy control speakers (mean age = 51.0, SD = 19.3) with no reported history of speech, language, or neurological impairment. Dysarthria was determined by an experienced speech-language pathologist and independently confirmed by a second experienced speech-language pathologist who was unaffiliated with the experiment. Speech intelligibility was assessed by the reading format of the *Assessment of Intelligibility of Dysarthric Speech* (Yorkston and Beukelman, 1984). Intelligibility scores ranged from 90 to 100%. Additional test batteries were used to screen speakers for dementia (*Arizona Battery of Communication Disorders of Dementia*; Bayles and Tomoeda, 1993), auditory processing impairments (*Revised Token Test*; McNeil and Prescott, 1978) and phonological processing deficits (*Psycholinguistic Assessment of Language Processing in Aphasia*; Kay et al., 1992). For descriptive purposes, participants also completed the digit span subtest of the Wechsler Adult Intelligence Scale to evaluate verbal working memory and the *Visual Patterns Test* (Della Sala et al., 1997) to estimate visual memory. Speakers with dysarthria required 2 days to complete the experiment. Cognitive, language, and speech testing was completed on the first day and the experimental task took place on the second day. The clinical characteristics of the dysarthric speakers are listed in **Table  1** for the participants with ataxic dysarthria and in **Table 2** for the participants with hypokinetic dysarthria. Speakers with hypokinetic dysarthria were evaluated under optimum medication per self-report. Additional information is available in Spencer and Rogers (2005). All of the patients in the hypokinetic dysarthria group had diagnoses of Parkinson’s disease except for two; participant H1 was diagnosed with multiple system atrophy (MSA) and participant H3 was diagnosed with corticobasal degeneration (CBD). The complexity of basal ganglia circuits (Parent and Hazrati, 1995) suggests that differences in basal ganglia pathology may affect outcomes during a maximum performance task and for this reason the data from the two non-Parkinson’s speakers with hypokinetic dysarthria were excluded from statistical analyses. Instead, informal comparisons between hypokinetic speakers with and without Parkinson’s disease were performed to gage differences in speech performance in speakers with perceptually similar speech disorders but different basal ganglia pathologies. In addition, the data from one healthy speaker (S002) were excluded from the current study as this speaker was diagnosed with amyotrophic lateral sclerosis approximately a year after participating in the Spencer and Rogers (2005) study.

**Table 1:** Clinical characteristics of participants with ataxic dysarthria.

Speaker	Age	Sex	Diagnosis	Duration (year)	Cerebellar signs			Dysarthria severity	Sentence intelligibility (%)
							
					Upper limbs	Gait	Oculomotor
A1	38	F	Cerebellar toxicity	10	–	+	–	Moderate	98
A2	22	F	Friedreich’s ataxia	8	+	+++	+	Mild	98
A3	27	F	Friedreich’s ataxia	13	++	+++	+	Moderate	90
A4	29	F	Friedreich’s ataxia	10	+	+++	+	Mild	99
A5	34	M	Unknown	1.5	+	+++	++	Moderate	96

*–, no impairment; +, mild impairment; ++, moderate impairment; +++, severe impairment.*

**Table 2:** Clinical characteristics of participants with hypokinetic dysarthria.

Speaker	Age	Sex	Diagnosis	Duration (year)	Basal ganglia signs			Dysarthria–dysphonia I	Sentence intelligibility (%)
							
					Tremor	Gait	Akinesia Bradykinesia
HI	66	F	Multisystem atrophy	6	–	++	+	Moderate	95
H2	78	F	Parkinson’s disease	3	–	+	+	Mild	100
H3	35	F	Corticobas al degenerati on	7	–	+++	+++	Moderate-severe	99
H4	67	M	Parkinson’s disease	2	+	+	+	Mild	98
H5	60	F	Parkinson’s disease	4	+	+	++	Mild	99
H6	58	M	Parkinson’s disease	2	+	–	–	Moderate	96
H7	77	F	Parkinson’s disease	2	+	–	–	Mild	99
H8	65	F	Parkinson’s disease	16	+	+	Dyskinesias	Mild	99
H9	84	M	Disease; essential tremor	5	++	+	+	Moderate	94
H10	51	F	Parkinson’s disease	11	–	+	Dyskinesias	Negligible	100

### STIMULI AND PROCEDURES

Syllables sequence stimuli from Experiment 2 of the Spencer and Rogers (2005) study are shown in **Table  3**. Speech production data of these stimuli were selected for analysis because the stimuli for that study consisted of heterogeneous syllable sequences that varied in length (between one and five syllables) and because the protocol yielded high error rates by speakers (mean error rate was 13% for control speakers, 12% for hypokinetic speakers, and 12% for ataxic speakers).

**Table 3:** Syllable sequence stimuli.

		Sequence length (in syllables)				
		
		1	2	3	4	5
Syllable combinations by sequence	1	ma	ma ka	ma ka na	ma ka na ha	ma ka na ha da
	2	da	da ha	da ha na	da ha na ka	da ha na ka ma
	3	ta	ta ma	ta ma ka	ta ma ka ha	ta ma ka ha na
	4	ja	ja cha	ja cha va	ja cha va za	ja cha va za tha
	5	va	va za	va za tha	va za tha sha	va za tha sha cha
	6	tha	tha sha	tha sha za	tha sha za va	tha sha za va cha

Syllable sequences were presented on a computer screen and subjects were instructed to produce each sequence as fast as possible, without compromising accuracy, and to produce the syllable sequence as a whole word. To ensure “whole-word” production during the experiment, a familiarization procedure was used before the experiment that consisted of: (1) a minimum of 120 practice trials; (2) two productions of each token with auditory and visual presentations; (3) two productions of each token with only visual presentation; and (4) extra practice sets were completed for frequent mispronunciations or consistent “segmenting” of the sequence (i.e., not reading the stimulus as a whole word).

Speech data were then acquired over five experimental runs of 60 trials each. At the beginning of each trial an alerter (cross) was displayed for 750 ms and, after 250 ms, the sequence was displayed and remained on the screen for 2000 ms. After a 1 s pause, the speech reaction time for the trial was displayed for the speaker to encourage vigilance and maintain rapid responding during the task. Sequence stimuli were displayed in black Courier New font (14–19 mm long, 13–15 mm wide) on a white background.

### ANALYSES

Speech acoustic data from the Spencer and Rogers (2005) study were recorded from DAT to PC at a sampling rate of 22050 Hz. Custom software routines were developed in Matlab (2011) to derive the *ISI* for each syllable in a sequence. Each ISI quantified the interval between the onset of one syllable and the onset of the following syllable. Because the current investigation addressed sequence selection, sequence-final syllables and one-syllable sequences were excluded from this analysis since selection processes are difficult to evaluate when there is only a single item in the response buffer.

Extraction of ISIs followed a two-stage process. The first stage was algorithmic and provided the user with automatically derived estimates of consonant onsets, vowel onsets, and vowel offsets based on the acoustic features of the sounds in a particular sequence. The second stage involved review of the automatically derived onset and offsets and corrections were applied as necessary by a trained user.

In the automated procedure, digital filters were applied to speech signal to isolate energy in spectral bands associated with different classes of sounds in a sequence. The filtered signals were converted to time series of dB values that quantified the magnitude of energy in each band over the course of the sequence. The dB time series were then convolved with a Gaussian derivative kernel to calculate their smoothed derivatives. The sampling rates of the dB time series and the width of the derivative kernel were adjusted to account for the different rates of spectral change associated with the onsets of sounds in different sound classes. Peaks in the smoothed derivatives were used to estimate vowel and consonant onsets in a sequence and valleys were used to estimate vowel offsets. Production of vowels was associated with spectral energy in the region of F1 (Howitt, 2000) and speech signals were digitally bandpass filtered using a fifth order Butterworth filter whose passbands corresponded to the minimum and maximum F1 frequencies produced by the speaker. The F1 minimum and maximum for each speaker were derived from a prior analysis of the F1 frequencies produced during the first 25 trials of the first and fifth runs. Spectral information was derived for the different consonants based on their manner of articulation. Nasal phonemes are characterized by predominately low frequency energy (Stevens, 1998), which was identified by digitally bandpass filtering the speech signal between 60 and 500 Hz. For stop consonants and affricates, the speech signal was high-pass filtered the speech signal at 2000 Hz. The resulting filtered signals for each sound class were converted to dB time series. Lastly, spectral energy associated with fricative production was derived from time series estimates of the first spectral moment calculated using a 12 ms window incremented in 4 ms steps through the speech signal. Smoothed derivatives of the dB time series were calculated. The parameters (e.g., sampling rates, window sizes) for deriving the dB time series and derivative signals for different phonemes were determined based on extensive pilot work based on the speech data from both healthy speakers and speakers with dysarthria.

The automated procedure identified the locations of vowel onsets and offsets first and, based on these locations, then identified the consonants onsets. Vowel onsets and vowel offsets for a *n*-syllable sequence were defined as the *n* largest peaks (onsets) that were separable by *n* valleys (offsets) in the vowel derivative trace. The algorithm was provided with the sequence for each trial and identified syllable onsets based on the consonants in each sequence. The *n*th consonant onset corresponded to the largest peak in the derivative trace of that consonant’s sound class that was located after the *n* - 1th vowel offset and before the *n*th vowel onset.

**FIGURE 1:**
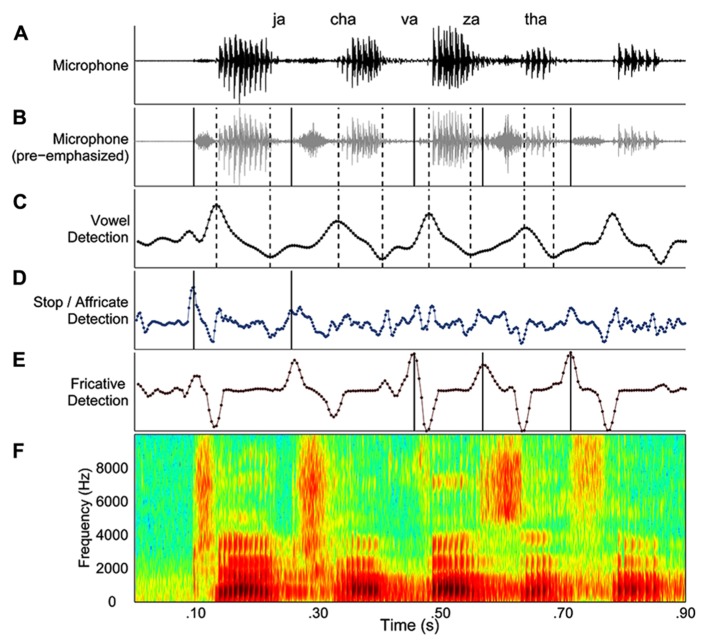
**An example of the graphical user interface for processing speech acoustic data from each trial.** **(A–E)** The display depicts the microphone signal **(A)**, the pre-emphasized microphone signal **(B)**, changes in spectral energy related to vowels **(C)**, stops and affricates **(D)**, and fricatives **(E)**. Lastly, the panel **(F)** depicts of a broadband spectrogram of the speech sequence produced by the speaker. Dashed lines indicate vowel onsets and vowel offsets in the “vowel detection” panel. Consonant onsets are indicated by solid lines in the panel corresponding to the manner of articulation for each consonant.

The second stage of the segmentation process involved a trained user’s review of the output of automated procedure. In this stage, a custom Matlab graphical user interface (GUI) displayed the automated segmentation results (**Figure 1**). The GUI included displays of that trial’s microphone signal (**Figure 1A**), pre-emphasized microphone signal (**Figure 1B**), the smoothed derivative for detecting vowels (Figure 1C), stops and affricates (**Figure 1D**), and fricatives (**Figure 1E**). **Figure 1F** displayed a broadband spectrogram of the speech sequence. The user reviewed automatically derived onsets and offsets by listening to the speech sequence, listening to the sequence segmented by syllable (playback imposed a 500 ms silence in between each syllable) and visually inspected the onsets and offsets against the spectrogram of the speech sequence. Errors in the automated segmentation routine were manually corrected, and then the onsets and offsets were re-plotted and the audio was re-segmented so the user could evaluate the accuracy of the corrections. On approximately 2% of the trials for two speakers with ataxia dysarthria, the identification of one or more syllables in a sequence was not possible. The consonant onsets in question were always obstruent consonants and were typically affricates. On these trials, the speaker’s production of the obstruent consonant exhibited the expected release/plosion that was followed by a brief silence and then a second consonant release/plosion was produced before production of the following vowel was initiated. As there was no basis for determining which release constituted the true onset of these consonant, it was impossible to calculate an ISI for these syllables and as a result these trials were excluded from the chronometric analyses. These trials were also excluded from the error analysis as these double-releases occurred quite rapidly in succession and were not detected during playback of the whole sequence, but only during playback of the segmented sequence and careful analysis of the spectrogram. These behaviors occurred on approximately 3% of the trials for one speaker and less than 1% of the trials for the other speaker. We speculate that these productions reflected articulatory incoordination, which is a common feature of ataxic dysarthria.

Analyses of sequence length and practice effects on speech errors were based on a system that scored sequences as accurate or as containing one or more error types. Speech errors were defined as productions that met any of the following criteria: (1) syllable/sound omission, insertion, transposition, or repetition, (2) initial syllable perseveration of previous target, (3) errors of consonant voicing, manner or place of articulation, (4) syllable segmentation/isolation, (5) self-corrections, and (6) unintelligible responses. Reliability was assessed conservatively using a strict agreement procedure, so that only identical coding of an error type (e.g., incorrect consonant place of articulation) was counted as an agreement. With this procedure, interjudge scoring agreement was found to be 93.8% for the control participants and 90.1% for the clinical participants. Trials containing errors were used to derive speech error rates associated with sequence length and practice. Counts of the number of trials containing an error were used to calculate a speaker’s mean error rate for each analysis. These data were then arcsine transformed since rate measures obtained from count data are typically not normally distributed (Winer et al., 1991). Trials containing errors were not included in analyses of speakers’ ISI data.

## RESULTS

### MEAN ISIs AND ERROR RATES BY GROUP

**FIGURE 2:**
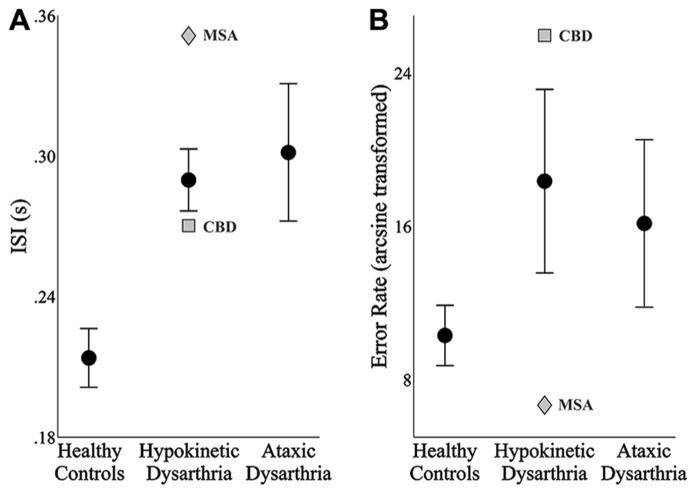
**The mean and standard error of the mean of each speaker group’s inter-syllable intervals (ISIs, A) and speech error rates (B)**. In the hypokinetic group, the means and standard error of the means were derived from the data of the hypokinetic speakers with Parkinson’s disease. In each plot, data from the hypokinetic speaker with multiple system atrophy (MSA, gray diamond) and the speaker with corticobasal degenera-tion (CBD, gray square) are indicated separately.

The average ISI and error rate (arcsine transformed) were derived for each speaker to characterize their overall speaking rate and accuracy during the reaction time task. **Figure 2** displays the mean and standard error of the means by group for speakers’ average ISIs (**Figure 2A**) and error rates (**Figure 2B**). Separate one-way ANOVAs were performed to test for main effects on group of average ISIs and error rates. Because this analysis consisted of two separate statistical tests, a Bonferroni correction was used to control for type I error and the adjusted *p*-value for significance was 0.05/2 = 0.025.

The results of the one-way ANOVA for ISIs identified significant differences across groups, *F*(2,24) = 9.54, *p* < 0.001. *Post hoc* testing with Bonferroni correction revealed that the average ISIs produced by the healthy speakers were significantly shorter than those produced by the speakers with hypokinetic dysarthria from Parkinson’s disease (*p* < 0.01, mean difference = 76 ms) and the speakers with ataxic dysarthria (*p* < 0.01, mean difference = 88 ms). No difference was detected in the ISIs between the speakers with hypokinetic and ataxic dysarthria. Levene’s test for equality of variance was found to be violated, *F*(2,24) = 6.37, *p* < 0.01, for the error rate by group analysis. As a result, group effects on speech error rates were evaluated using a Kruskal–Wallis test. The results of this test did not identify a significant effect of group on error rate, χ^2^(2, *N* = 27) = 2.67, *p* = 0.263.

The average ISIs and error rates for the speakers with hypokinetic dysarthria and CBD (depicted with a gray square in **Figure 2**) and MSA (depicted with a gray diamond in **Figure 2**) tended to fall outside the standard error of the means for the speakers with hypokinetic dysarthria with Parkinson’s disease. For both speakers, a trade-off between ISIs and error rates, albeit in opposite directions, was observed with the MSA speaker exhibiting a slower speaking rate with fewer errors and the CBD speaker exhibiting a faster speaking rate with more errors. The trade-off was especially prominent for the speaker with CBD.

### SEQUENCE LENGTH EFFECTS ON INTER-SYLLABLE INTERVALS

Mean ISIs were derived at each of the four sequence lengths for each speaker. Separate linear mixed effects models were used to test for main effects of sequence length on ISIs in each group. In these models, a significant fixed effect of length consisted of an intercept term, which describe the “baseline” ISI at a sequence length of two, and a slope term, which described the rate of change of speakers’ ISIs with each syllable increase in sequence length. Together, these fixed effects coefficients characterized the intercept and slope of the line of the best fit describing ISI by length effects. The random effect corresponded to speaker-specific differences in the intercept term of the model’s fits to each speaker’s data. To control for type I errors associated with multiple statistical tests (one for each group), the *p*-threshold for significance was set to 0.05/3 = 0.0167.

**FIGURE 3:**
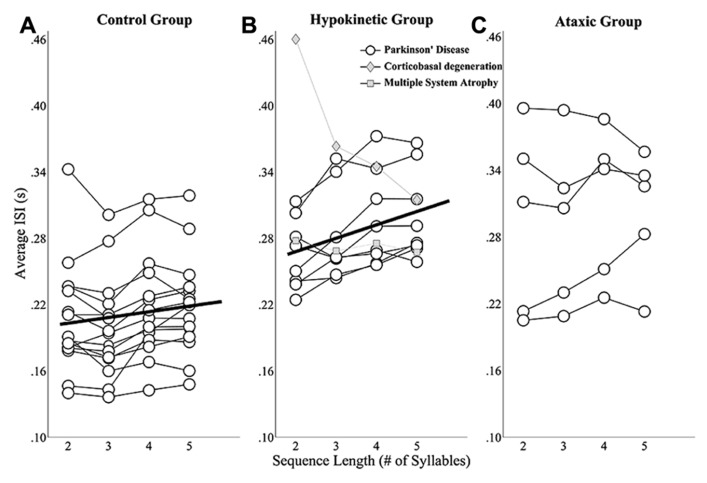
**Sequence length effects on average ISIs for the healthy speaker group (A)**, the hypokinetic speaker group **(B)**, and the ataxic speaker group **(C)**. Linear mixed effects models revealed significant effects of sequence length on average ISIs in the healthy speakers and hypokinetic speakers with Parkinson’s disease. The line of best fit is displayed in the plots for these groups. Data from the hypokinetic speaker with MSA (gray diamonds) and the speaker with CBD (gray squares) are indicated separately in the plot for the hypokinetic speakers **(B)**. No significant effect of sequence length on the average ISIs of the ataxic speakers was observed.

The mixed effects analysis of healthy speakers’ ISIs revealed a significant effect of sequence length *t*(41) = 2.76, *p* < 0.0167). The slope coefficient describing the relationship between sequence length and ISIs was 4.9 indicating that speakers’ average ISIs increased ~5 ms for each additional syllable in a sequence. These effects are depicted in **Figure 3A**, which shows speakers’ individual ISI data as well as the line of best fit for the group. A significant main effect of sequence length on ISIs was also observed for the speakers with hypokinetic dysarthria from Parkinson’s disease, *t*(23) = 5.43, *p* < 0.05 and, for these speakers, average ISIs increased by 12 ms for each additional syllable (**Figure 3B**). The linear mixed effects model failed to identify a significant effect of sequence length on mean ISIs in the speaker with ataxic dysarthria, *t*(14) = 1.06, *p* > 0.05. The results for the speakers with ataxia are depicted in **Figure 3C** without a line of best fit to denote the lack of a statistical relationship between speakers’ average ISIs and sequence length.

These findings revealed that healthy speakers and speakers with hypokinetic dysarthria from Parkinson’s disease exhibited the expected effects of sequence length on ISIs but that this effect was not present in the speakers with ataxic dysarthria. In addition, the finding of a larger slope term in the hypokinetic group suggested that speakers in this group exhibited a larger effect of sequence length on ISIs than speakers in the healthy group. To evaluate this possibility, data from the healthy and Parkinson’s groups were combined and a mixed effects analysis evaluated the main and interaction effects of sequence length and group on mean ISIs. In this model, the interaction term evaluated whether the slopes of the fits for the two groups were significantly different. Since the main effects for the individual groups were reported above, only the interaction effects in this test are reported here. The interaction of sequence length and group was found to be significant, *t*(64) = 2.52, *p* < 0.05, and revealed a significantly higher slope in the fit for the speakers with hypokinetic dysarthria compared to the healthy speakers. The magnitude of this slope increase in the speakers with hypokinetic dysarthria was 7 ms per syllable.

In the length by ISI analysis, the hypokinetic speaker with MSA and the hypokinetic speaker with CBD appeared to distinguish themselves both from the speakers with hypokinetic dysarthria from Parkinson’s disease as well as from each other. The length by ISI function for the speaker with appeared a bit flatter than that of any of the speakers Parkinson’s disease. Again, the more profound difference was exhibited by the speaker with CBD who demonstrated a robust effect of sequence length on ISIs that was in the direction opposite that of the healthy speakers and the speakers with Parkinson’s disease.

### SEQUENCE LENGTH EFFECTS ON SPEECH ERRORS

**FIGURE 4:**
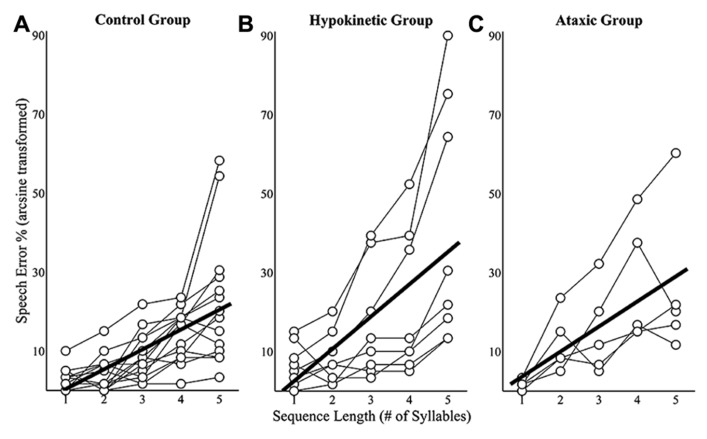
**Sequence length effects on speech error rates for the healthy speaker group (A)**, the hypokinetic speaker group **(B)**, and the ataxic speaker group **(C)**. Significant effects were observed in the healthy speaker group and hypokinetic group with Parkinson’s disease. The line of best fit is displayed in the plots for these groups.

Sequence length effects on speech error rates were examined using mixed effects models that evaluated speakers’ mean error rates at each of the five sequence lengths. Speech error rates for a given sequence length were derived by dividing the number of errors produced by a speaker by 60 (i.e., the number of trials produced at each sequence length) and arcsine transforming the results. This analysis revealed a sequence length effect on speech error rates in the healthy speaker group, *t*(55) = 8.41, *p* < 0.0167, the hypokinetic speaker group, *t*(31) = 6.07, *p* < 0.0167, and the ataxic speaker group, *t*(19) = 5.32, *p* < 0.0167. An examination of the slope coefficients revealed that each syllable increase in sequence length was associated with a 5.0% increase in error rates in the healthy speaker group, an 8.2% increase in the hypokinetic group, and a 6.3% increase in the ataxic speaker group. These effects of sequence length on speakers’ mean error rates are displayed in **Figure 4**. Data are displayed in **Figure 4A** for the healthy speakers, in **Figure 4B** for the hypokinetic speakers with Parkinson’s disease, and in **Figure 4C** for the speakers with ataxic dysarthria. The error rate by length functions for the hypokinetic speaker with CBD and the hypokinetic speaker with MSA were comparable to those of the speakers with hypokinetic dysarthria from Parkinson’s disease. As a result, their data have been omitted from **Figure 4** to simplify the display of information.

Speech error rates from all three groups were combined and a linear mixed effects analysis was performed to evaluate the main and interaction effects of group and sequence length speech error rates. This analysis identified a significant interaction effect between length and group on speech error rates, *t*(105) = 2.48, *p* < 0.05. *Post hoc* comparisons revealed that the error rates of the speakers with hypokinetic dysarthria occurred at a significantly faster rate (*p* < 0.0167, mean difference = 3.2%/per syllable) than those of healthy speakers. No significant interaction effects were observed in the comparison of speakers with ataxic dysarthria to healthy speakers (*p* = 0.38) and speakers with dysarthria (*p* = 0.26). In summary, this analysis revealed that the speech error rates of all three speaker groups increased with increases in sequence length but that the rate of length-dependent increases in speech errors was significantly larger in the group with hypokinetic dysarthria than in either of the other two groups.

### PRACTICE EFFECTS ON INTER-SYLLABLE INTERVALS

**FIGURE 5:**
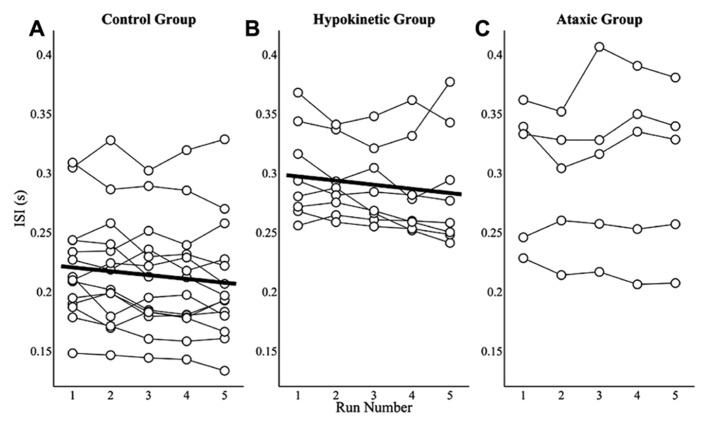
**The effects of practice on average ISIs are depicted by run for the healthy speaker group (A)**, the hypokinetic speaker group **(B)**, and the ataxic speaker group **(C)**. Significant effects were observed in the healthy speaker group and hypokinetic group with Parkinson’s disease but not in the ataxic speaker group.

Practice effects on ISIs were assessed by averaging speakers’ ISIs for each of the five runs to evaluate changes in average ISIs over the course of the study. Mixed effects analyses revealed that the healthy speaker group exhibited a small but significant decrease in mean ISI, *t*(55) = -3.52, *p* < 0.0167, of 3.2 ms per run. The mean ISIs by run and model fit for the healthy speaker group are shown in **Figure 5A**. A similar effect of practice was observed for the hypokinetic speakers with Parkinson’s disease, *t*(31) = -2.59, *p* < 0.0167. This group exhibited an average decrease in mean ISIs of 3.5 ms per run (see **Figure 5B**). A significant effect of practice on ISIs was not observed for the speakers with ataxic dysarthria, *t*(19) = 0.91, *p* = 0.38 (see **Figure 5C**). A combined analysis of the ISIs from the healthy and hypokinetic groups did not reveal a significant interaction between group and run, *t*(86) = -0.19, *p* = 0.85, indicating that the run-dependent decreases in ISIs for these two groups were not significantly different. Inspection of the findings for the speakers with MSA and CBD suggested that the effects of run on average ISIs was comparable to that observed for the speakers with hypokinetic dysarthria from Parkinson’s disease. For this reason, the data from speakers with MSA and CBD have been omitted from **Figure 5**.

### PRACTICE EFFECTS ON SPEECH ERRORS

Speakers’ error rates (arcsine transformed) for each run were derived to evaluate the effects of practice on speech error rates. A significant effect of practice on speech error rates was observed for the healthy speaker group, *t*(55) = -3.54, *p* < 0.0167, with this group exhibiting an average decrease in error rate of 1.1% per run. The speakers with hypokinetic dysarthria also exhibited a practice effect, *t*(31) = -4.25, *p* < 0.0167, and the error rates in this group decreased by an average of 1.9% per run. Practice effects on speech error rates were not observed in the group of speakers with ataxic dysarthria, *t*(19) = 0.75, *p* = 0.47. A combined analysis of the speech error data of the healthy and hypokinetic speaker groups failed to identify a significant interaction between run and group, *t*(86) = -1.52, *p* = 0.13. This finding indicated that the practice-related decreases in error rates were not significantly different between these two groups. Visual inspection of the speech data for the speakers with MSA and CBD suggested that these speakers exhibited run-related decreases in error rates that were comparable those of hypokinetic speakers with Parkinson’s disease.

## DISCUSSION

The current study investigated the effects of sequence length and practice on within-sequence measures of speech performance in healthy speakers and speakers with hypokinetic or ataxic dysarthria. Analyses of speech sequence performance revealed different effects of sequence length on the hypokinetic speakers with Parkinson’s disease and the speakers with ataxic dysarthria. Compared to the healthy speaker group, speakers with hypokinetic dysarthria exhibited significantly larger sequence length effects on both ISIs and speech error rates, which is consistent with an action selection impairment during sequence production. In contrast, speakers with ataxic dysarthria failed to demonstrate an effect of sequence length on ISIs, which is consistent with a deficit in buffering to-be-produced speech items prior to sequence production. In addition, the speakers with ataxic dysarthria failed to exhibit an effect of practice on either ISIs or speech error rate. This latter finding suggests that learning-related increases in speech rate and accuracy incorporates processing by the cerebellum.

### SERIAL CONTROL IN HEALTHY SPEAKERS

In the current study, the healthy control speakers exhibited significant increases in average ISIs with increases in sequence length. This finding is consistent with those of Sternberg et al. (1978) and Klapp (2003) and demonstrate that, in addition to the well-documented effects of sequence length on speech reaction times (Klapp, 1974; Sternberg et al., 1978, 1980; Peters et al., 1989; Jared and Seidenberg, 1990; Deger and Ziegler, 2002; Roelofs, 2002; Santiago et al., 2002; Spencer and Rogers, 2005), sequence length also influences the selection of speech items *during* speech sequence production. Increases in sequence length were also associated with increased speech error rates in the healthy speaker group. Although not extensively studied, a sequence length effect on speech error rates is suggested by previous speech reaction time findings. For example, Sternberg et al. (1980) reported negligible error rates during production of randomly ordered lists of digits and numbers containing five items or less, but considerably higher error rates during production of lists containing six items. In addition, Jared and Seidenberg (1990) observed higher error rates during production of *n* + 1 syllable words compared to *n* syllable words for words containing between one and four syllables. A similar association between error rates and sequence length has also been observed during immediate serial recall tasks (Drewnowski and Murdock, 1980). Several investigators have suggested that the similarity of error patterns observed during speech production and immediate serial recall suggest that serial short-term memory and serial control for speech share a common processing mechanism (Ellis, 1980; Page et al., 2007) and the current findings support this proposal.

Significant practice-related decreases in mean ISIs (equivalent to increases in speaking rate) and error rates were identified in the healthy speaker group as well as in the group with hypokinetic dysarthria from Parkinson’s disease. The magnitude of these effects was, however, quite limited. Healthy speakers’ average ISIs decreased by approximately 13 ms from the first to the fifth run while the ISIs of speakers with Parkinson’s disease decreased by approximately 14 ms. Speech error rates exhibited similarly small decreases. The magnitude of practice-related changes may have resulted from the relation of different speech sequences to each other. Specifically, speech stimuli consisted of six basic sequences with each sequence containing a particular combination of consonant–vowel syllables. To evaluate sequence length effects, each of the six different sequences was presented with equal frequency at one of five sequence lengths. For example, the stimulus, “ma ka na ha da” was presented as often as the stimuli, “ma,” “ma ka,” “ma ka na,” and “ma ka na ha.” As a result, the order of the syllables in a particular sequence was not fixed but depended on the length of the sequence being presented. An arrangement whereby early items in a sequence are followed by a variable number of subsequent items bears some similarity to lexical and sentence constructions for spoken language. At the same time, this arrangement of sequences limits the efficiency that can be gained by chunking consecutive syllables within a sequence and suggests that constraints on chunk learning contributed to the limited practice effects in the present study. That some syllables were presented in more than one sequence would have posed an additional constraint on chunk learning.

Although the magnitude of practice effects was small, their significance is notable in that it suggests a role for context-dependent item-by-item learning sequence practice. Although this type of learning has not been extensively investigated, Rhodes and Bullock (42, 100) and Rhodes and colleagues (30) have posited that, because context-dependent learning is derived from multiple sources of information about a sequence, this type of sequence learning is well-suited to learning “different branches from the stem” (42), such as the sequences in the present study. In addition, Rhodes and Bullock (Rhodes and Bullock, 2002a, b; Rhodes et al., 2004) have claimed that the effects of context-dependent learning would be most evident after extensive practice. Since sequence performance was only evaluated over a single session, the limited effects of practice are consistent with the purported contribution of this type of learning to the current findings.

### SERIAL CONTROL IN SPEAKERS WITH HYPOKINETIC DYSARTHRIA FROM PARKINSON’S DISEASE

Despite the well-documented role of the basal ganglia in action selection, there has been little consensus in the research literature regarding serial control functioning in patients with Parkinson’s disease. For example, Connor et al. (1989) observed similar speech error and production rates in speakers with dysarthria from Parkinson’s disease and healthy control speakers during production of one and two-syllable sequences repeated as quickly as possible. The investigators concluded from these results that speech accuracy and timing are not affected by the length or complexity of a speech utterance in Parkinson’s disease. These findings are consistent with those of Rafal et al. (1987) who investigated sequence length effects in the patients with Parkinson’s disease and healthy participants during a keypress task. Sequences were between one and three keypresses in length and the authors reported longer reaction times and inter-response intervals in the Parkinson’s patients, but found that the effects of sequence length in these patients were comparable to those produced by healthy participants (i.e., they observed no differences in the slopes describing sequence length and inter-response intervals between the healthy controls and the group with Parkinson’s disease).

However, a similar study by Agostino et al. (1992) produced a different result. These investigators evaluated inter-response intervals during production of sequential arm movement sequences that were either two, three, four, or five arm movements in length. Participants were instructed to perform the sequences as fast as possible. Like Rafal et al. (1987), Agostino et al. (1992) found that individuals with Parkinson’s disease produced longer inter-response intervals, but observed no difference in slope values during production of sequences containing two or three arm movements. However, their analysis revealed that the slope values during production of sequences that were either four or five arm movements in length were larger for the patients with Parkinson’s disease than the healthy control participants. This set of findings was unique to the group with Parkinson’s disease in this study as increased slope values were not observed for patients with Huntington’s disease or patients with dystonia. These findings by Agostino et al. (1992) might account for the discrepancy between the current findings and those of Connor et al. (1989) and Rafal et al. (1987). Specifically, the present findings as well as those of Agostino et al. (1992) provide evidence for an action selection deficit causing exaggerated sequence length effects in patients with Parkinson’s disease, but suggest that this deficit is only detectable during production tasks that involve production of both shorter and longer sequences.

In contrast to speakers in the ataxic group, speakers in the group with Parkinson’s disease exhibited larger sequence length effects than the healthy control group. The exaggerated length effects of the group with Parkinson’s disease cannot be attributed to their slower speaking rate, since speakers with ataxic dysarthria exhibited similarly slow speaking rates, or to fatigue effects, since the average ISIs and error rates of speakers with disease Parkinson’s disease decreased over the course of the study. Instead, the exaggerated length effects are consistent with an action selection impairment in speakers with Parkinson’s disease that is characterized by longer intervals to select an upcoming action as well as higher likelihoods of a selection error. This type of impairment might account for a number of speech symptoms, including inappropriate pauses, disfluencies, and difficulties initiating speech, that are commonly associated with hypokinetic dysarthria in speakers with Parkinson’s disease (Svensson et al., 1993; Sapir et al., 2008).

The increased ISIs and error rates in the speakers with Parkinson’s disease are consistent with the basal ganglia’s role in facilitating action selection and with the effects of dopamine on this process. Specifically, dopamine in the striatum is believed to facilitate selection by increasing the contrast between the activation level of a high priority action and those of competing, lower priority actions (Foote and Morrison, 1987; Cohen and Servan-Schreiber, 1992; Kiyatkin and Rebec, 1996; Rebec, 1997; Boraud et al., 2000; Cohen et al., 2002; Leblois et al., 2006). In the context of serial control, the high priority action corresponds to the to-be-produced-next item in a sequence (Bullock and Rhodes, 2003; Rhodes et al., 2004). The contrast reduction associated with dopamine depletion increases the amount of time to a select a particular item, and also decreases the likelihood of correctly selecting a particular item. A selection impairment of this type might also account for two commonly cited deficits in Parkinson’s patients: response switching (Weiss et al., 1997; Inzelberg et al., 2001; Praamstra and Plat, 2001; Rubchinsky et al., 2003) and response maintenance (Jahanshahi et al., 1993; Gueye et al., 1998; Gentilucci and Negrotti, 1999a, b). Difficulty switching between responses corresponds to the reduced contrast between item activations that increases the time to select an item when other competing or distractor items are present. Difficulties with response switching, in turn, prolong the duration of a sequence and, as a result, increase the adverse effects of either decay (Page and Norris, 1998) or interference (Oberauer et al., 2012) on maintenance of item activations currently in the buffer.

Speech findings from two speakers with hypokinetic dysarthria and diagnoses of CBD and MSA suggested that these speakers’ sequence performance differed from the performance of speakers with hypokinetic dysarthria from Parkinson’s disease who had similar diagnoses and speech perceptual characteristics. For both speakers, qualitative differences in speech rate, error rate, and sequence length effect on average ISIs appeared to distinguish these speakers with hypokinetic dysarthria from the speakers with hypokinetic dysarthria from Parkinson’s disease. In addition, the observed differences tended to distinguish the two speakers from each other. For example, the average ISI of the speaker with MSA fell within the bottom of half of the ISIs produced by the speakers with Parkinson’ disease but the average ISI of the CBD speaker was equal to the largest value observed in the Parkinson’s group. At the same time, the CBD speaker exhibited an error rate that was among the lowest produced by the speakers with Parkinson’s disease, but the error rate of the MSA speaker was in the upper half of the error rates produced by the Parkinson’s group. In addition, the speaker with MSA did not appear to exhibit a sequence length effect on ISIs but the speaker with CBD exhibited a strong length effect on ISIs. However, the direction of the sequence length effect in the speaker with CBD was opposite that of speakers with Parkinson’s disease and also opposite that of healthy speakers documented in the research literature. Specifically, the average ISI for this speaker’s two-syllable sequences was nearly double that of the speakers with Parkinson’ disease and then decreased in a roughly linear fashion as the number of syllables in the sequence increased but was still relatively high during production of five-syllable sequences. The findings of the MSA and CBD speakers cannot be regarded as representative in any sense, but they do suggest that differences in basal ganglia neuropathology are associated with qualitatively different impairments in serial control for speech. Of all the analyses, the effects of sequence length on average ISIs appeared to be most diagnostically useful as this analysis differentiated the three experimental groups from each other and also appeared to distinguish the speakers with CBD and MSA from the speakers with Parkinson’s disease and from each other.

### SERIAL CONTROL IN SPEAKERS WITH ATAXIC DYSARTHRIA

Speakers with ataxic dysarthria failed to demonstrate the expected increases in mean ISIs associated with increases in sequence length. The current findings extend those of Spencer and Rogers (2005) who observed reduced sequence length effects on speech reaction times in this same group of speakers. The findings from these two studies are consistent with those of Inhoff et al. (1989) who observed that moderately ataxic patients failed to demonstrate sequence length effects on reaction times and inter-response intervals during a sequential keypress task. Together, the results of these investigations indicate that cerebellar diseases may impair the pre-loading of to-be-produced sequence items into a response buffer and, as a result, not all items in the sequence are available at the time of sequence initiation. Instead, items are loaded *during* sequence production and executed as “functionally separate unit(s)” (Inhoff et al., 1989). The common finding of a “scanning” speech quality in speakers with ataxic dysarthria (Kent et al., 2000) suggests such a functional separation of units during speech production and raises the possibility that this prominent characteristic of ataxia dysarthria is attributable to a deficit in pre-loading items in a speech utterance. Kent et al. (1979) reached a similar conclusion in their acoustic analysis of speakers with ataxic dysarthria. These investigators noted that the speech timing and prosodic characteristics of ataxic speakers suggested that syllables were not integrated within longer sequences or phrases. Rather, syllables appeared to be produced as single units and that this quality of their speech likely reflected a reduced capacity to buffer longer speech responses.

The notion that the cerebellum, through its connections with cortex, is involved in buffering verbal information has received considerable support from both neuroimaging and clinical investigations. In their neuroimaging study of speech sequence production, Bohland and Guenther (2006) observed two main areas of activation in the cerebellum. One focus of activation was in the right inferior cerebellum, which exhibited a main effect for sequence complexity (i.e., sequences of the same versus different syllables). The second focus was a moderately right lateralized area in the superior cerebellum that responded to syllable complexity (i.e., syllable sequences that contained a single consonant followed by a vowel versus syllable sequences that contained two or three consonants followed by a vowel) as well as the interaction of sequence and syllable complexity. These findings indicate that the cerebellum is involved in the motor programing of speech sequences and not just their execution. These regions of cerebellum have also been associated with load-dependent changes in activation during verbal working memory tasks (Desmond et al., 1997; Chen and Desmond, 2005; Kirschen et al., 2005; Marvel and Desmond, 2010a, 2012). In addition, damage to the cerebellum has been associated with a number of verbal working memory deficits (Malm et al., 1998; Silveri et al., 1998; Ravizza et al., 2006; Chiricozzi et al., 2008; Misciagna et al., 2010). Together, these findings indicate that the cerebellum is part of a network of working memory buffers that are engaged in serial processing for speech (Ackermann et al., 2004; Spencer and Slocomb, 2007). Damage to the cerebellum could impair the functioning of these buffers and, in turn, limit the buffering of to-be-produced speech items as suggested by the current and previous findings (Inhoff et al., 1989; Spencer and Rogers, 2005).

Although the ataxic speakers did not exhibit sequence length effects on ISIs, this group did demonstrate a significant effect of sequence length on speech error rates. The dissociation between chronometric and error findings in this analysis was the only instance in this study where chronometric and error results were not identical. The reason for this discrepancy between length effects on ISIs and error rates is not clear, but may have been a consequence of partial buffering of sequence items; a finding that is suggested by the reduced, but not absent, effects of sequence length on speech reaction times previously reported for these speakers (Spencer and Rogers, 2005). It may be that items loaded during sequence production are more prone to error than ones loaded prior to production. In this case, pre-loading a portion of the items in a sequence may be sufficient to produce sequence length effects on error rates, but not on ISIs.

In contrast to both the healthy and Parkinson’s speaker groups, the ataxic speaker group failed to demonstrate practice-related decreases in average ISIs (i.e., increases in speech rate) and error rates over the course of the experiment. It is difficult to gage the import of this negative finding given the small sample of ataxic speakers and the magnitude of the practice effects in the healthy and Parkinson’s groups. Nevertheless, the finding that the latter groups exhibited practice-related decreases for both ISIs and error rates but that neither measure was significant in the ataxic group indicates an impairment in practice-related processing in these speakers. In addition, this finding is consistent with those of previous studies reporting sequence learning deficits in cerebellar patients (Pascual-Leone et al., 1993; Molinari et al., 1997; Gomez-Beldarrain et al., 1998) and is in agreement with the demonstrated role of the cerebellum in sequence learning (Jenkins et al., 1994; Toni et al., 1998; Steele and Penhune, 2010) and retrieval of learned sequences (Lu et al., 1998; Wu et al., 2008; Steele and Penhune, 2010). In particular, Rhodes and Bullock (2002a), Durisko and Fiez (2010), and Rhodes et al. (2004) have proposed a module for learning item-by-item transitions in a sequence, which we suggest is responsible for the practice effects observed in the present study, that is located in the inferior cerebellum. Damage to this learning module and/or its input or output pathways could account for the absence of practice effects in the speakers with ataxic dysarthria. Although the finding of both speech and non-speech studies point toward reduced practice effects in patients with ataxia, it is also possible that fatigue also contributed to the absent practice effects in speakers with ataxic dysarthria (Vogel et al., 2011; Vogel and Maruff, 2013).

### SERIAL CONTROL IN NEUROLOGIC POPULATIONS

Traditional descriptions of the speech characteristics of speakers with either ataxic or hypokinetic dysarthria have tended to focus on neuromuscular or motor execution deficits in these speakers. In the current study, both the hypokinetic and ataxic dysarthria groups exhibited slower speaking rates than the healthy group; however no difference in speaking rate was observed between the two groups with dysarthria. Reduced rate of speech is a characteristic feature of most types of dysarthria, including ataxic and hypokinetic dysarthria. A relevant question is whether the decreases in speaking rate observed in these populations was attributable to serial control. As noted, the analysis of sequence length effects revealed that the dysarthria groups differed not only from the healthy controls, but also from each other: sequence length effects were absent in the ataxic group, but exaggerated in the group with Parkinson’s disease. Despite these differences, the length effects exhibited by both groups would have contributed to their overall slower speaking rates. For example, pre-loading allows for faster production of speech sequences since the to-be-produced items are already buffered and available for selection (Rhodes et al., 2004). Limited pre-loading of the response buffers in the cerebellar speakers reduces speech rate because of the processing delays introduced by the need to continually load new items into the buffer. In the Parkinson’s group, the larger length effects on ISIs resulted in longer ISIs during intermediate and long sequences. It follows, then, that overall speaking rates would have been faster (i.e., associated with shorter mean ISIs) in the absence of an increased length effect. To summarize, both dysarthria groups exhibited sequence production deficits that reduced speaking rate but these deficits reflected quite different impairments in serial control. The finding that a portion of the slow speaking rates could be accounted for by different impairments in serial control raises the possibility that the generalized slowing of speech rates in dysarthria may be attributable to factors that are specific to each type of dysarthria, and that these factor can include motor programing impairments related to speech sequencing in addition to motor execution impairments.

## Conflict of Interest Statement

The authors declare that the research was conducted in the absence of any commercial or financial relationships that could be construed as a potential conflict of interest.
